# Tying the knot: An uncommon complication

**DOI:** 10.1002/ccr3.2279

**Published:** 2019-06-24

**Authors:** Konstantinos Boumpoulis, Andrew C. Chatzis, Marina Balanika, Michael Milonakis, Mazen Khoury

**Affiliations:** ^1^ 2nd Department of Cardiac Surgery Onassis Cardiac Surgery Centre Athens Greece; ^2^ Department of Anaesthesia Onassis Cardiac Surgery Centre Athens Greece

**Keywords:** complications, entangled indwelling catheter, pulmonary artery catheter, Swan‐Ganz

## Abstract

Cardiothoracic procedures require continuous hemodynamic monitoring and a fair proportion of these require the insertion of a pulmonary artery catheter, known also as Swan‐Ganz catheter. Given, however, the invasive nature of these procedures, unforeseen complications may ensue. Early recognition and appropriate handling are essential to minimize adverse outcomes.

A 40‐year‐old man underwent uneventful elective cardiac surgery (CABG) for coronary artery disease. Central venous access achieved via the right subclavian approach, included both a central venous and a pulmonary artery catheter (PAC). Immediate postoperative chest x‐ray revealed self‐entanglement of the PAC (Figure 1). The catheter was successfully removed by means of a minor vascular procedure.

**Figure 1:**
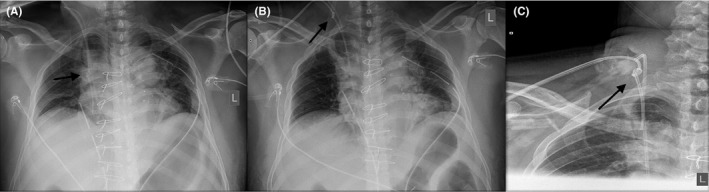
Pulmonary artery catheter (PAC) knot at the original discovery position (A), retrieved to the entry (removal) point (B), and closer view (C)

A certain number of patients with PAC experience complications. Complications may arise at any step of the procedure, from central venous puncture to catheter insertion and of course during their presence inside the patient. These include accidental arterial puncture, hematomas, thrombosis and embolism, arrhythmias, air embolization, infections, even right ventricular free wall, and pulmonary artery rupture. Although entanglement with other catheters such as central venous lines indwelling in the same vein is well described, pulmonary artery catheter knotting is extremely rare (0.03%). Nonetheless, pulmonary catheters are more likely to knot due to their length and flexibility. Surgical removal is the only safe option.^1^, ^2^

## CONFLICT OF INTEREST

None declared.

## AUTHOR CONTRIBUTION

KB: collected the data and drafted the article. ACC: conceived the study, analyzed and interpreted the data, and revised the article critically for important intellectual content. MB, MM: collected the data. MK: approved the final version of the manuscript.
